# Hesitancy for receiving regular SARS-CoV-2 vaccination in UK healthcare workers: a cross-sectional analysis from the UK-REACH study

**DOI:** 10.1186/s12916-022-02588-7

**Published:** 2022-10-10

**Authors:** Neyme Veli, Christopher A. Martin, Katherine Woolf, Joshua Nazareth, Daniel Pan, Amani Al-Oraibi, Rebecca F. Baggaley, Luke Bryant, Laura B. Nellums, Laura J. Gray, Kamlesh Khunti, Manish Pareek, The UK-REACH Study Collaborative Group, Anna L. Guyatt, Catherine John, I. Chris McManus, Ibrahim Abubakar, Amit Gupta, Keith R. Abrams, Martin D. Tobin, Louise Wain, Sue Carr, Edward Dove, David Ford, Robert Free

**Affiliations:** 1 Department of Respiratory Sciences, University of Leicester, Leicester, UK; 2 Department of Infection and HIV Medicine, University Hospitals of Leicester NHS Trust, Leicester, UK; 3 University College London Medical School, London, UK; 4 Population and Lifespan Sciences, School of Medicine, University of Nottingham, Nottingham, UK; 5 Biostatistics Research Group, Department of Health Sciences, University of Leicester, Leicester, UK; 6 Diabetes Research Centre, University of Leicester, Leicester, UK

**Keywords:** Healthcare, Ethnicity, SARS-CoV-2, COVID-19, Vaccination, Hesitancy

## Abstract

**Background:**

Regular vaccination against SARS-CoV-2 may be needed to maintain immunity in ‘at-risk’ populations, which include healthcare workers (HCWs). However, little is known about the proportion of HCWs who might be hesitant about receiving a hypothetical regular SARS-CoV-2 vaccination or the factors associated with this hesitancy.

**Methods:**

Cross-sectional analysis of questionnaire data collected as part of UK-REACH, a nationwide, longitudinal cohort study of HCWs. The outcome measure was binary, either a participant indicated they would definitely accept regular SARS-CoV-2 vaccination if recommended or they indicated some degree of hesitancy regarding acceptance (probably accept or less likely). We used logistic regression to identify factors associated with hesitancy for receiving regular vaccination.

**Results:**

A total of 5454 HCWs were included in the analysed cohort, 23.5% of whom were hesitant about regular SARS-CoV-2 vaccination. Black HCWs were more likely to be hesitant than White HCWs (aOR 2.60, 95%CI 1.80–3.72) as were those who reported a previous episode of COVID-19 (1.33, 1.13–1.57 [vs those who tested negative]). Those who received influenza vaccination in the previous two seasons were over five times less likely to report hesitancy for regular SARS-CoV-2 vaccination than those not vaccinated against influenza in either season (0.18, 0.14–0.21). HCWs who trusted official sources of vaccine information (such as NHS or government adverts or websites) were less likely to report hesitancy for a regular vaccination programme. Those who had been exposed to information advocating against vaccination from friends and family were more likely to be hesitant.

**Conclusions:**

In this study, nearly a quarter of UK HCWs were hesitant about receiving a regular SARS-CoV-2 vaccination. We have identified key factors associated with hesitancy for regular SARS-CoV-2 vaccination, which can be used to identify groups of HCWs at the highest risk of vaccine hesitancy and tailor interventions accordingly. Family and friends of HCWs may influence decisions about regular vaccination. This implies that working with HCWs and their social networks to allay concerns about SARS-CoV-2 vaccination could improve uptake in a regular vaccination programme.

**Trial registration:**

ISRCTN Registry, ISRCTN11811602.

**Supplementary Information:**

The online version contains supplementary material available at 10.1186/s12916-022-02588-7.

## Background

The COVID-19 pandemic has caused economic, societal and public health disruption of an unprecedented magnitude [1]. Globally, vaccine programmes have been effective at preventing symptomatic disease [2], resulting in a reduced rate of hospitalisations and deaths [3]. In many countries, healthcare workers (HCWs) represent a priority group for vaccination due to their increased risk of COVID-19 compared to the general population and also to weaken the chain of transmission to vulnerable patient groups [4–6]. However, the emergence of variants which are less susceptible to vaccines, waning immunity and breakthrough infections has meant that COVID-19 continues to cause disruption, despite good vaccine uptake in the UK [7, 8]. As we transition through the pandemic, vaccination is of paramount importance in the UK where restrictions, including legal requirements for case isolation, have ended.

The highly transmissible B.1.1.529 omicron variant of SARS-CoV-2 could infect those who had received two doses of vaccine [9, 10]. The urgency for delivery of a third vaccine dose to establish protection against omicron was recognised as the UK, and other higher economic countries, embarked upon the booster phase of vaccination. For HCWs, in particular, it is increasingly likely that further doses of vaccine will be offered as UK policy has shifted towards treating SARS-CoV-2 as an endemic virus [11, 12]. Indeed, the UK’s Joint Committee on Vaccination and Immunisation recently announced that a further dose of SARS-CoV-2 vaccine should be delivered to ‘frontline’ HCW in Autumn 2022 [13].

Vulnerable groups in the UK were invited to receive a second SARS-CoV-2 booster vaccination in spring 2022, and there is a growing expectation that an annual SARS-CoV-2 vaccination will be offered to protect hospitals from expected COVID-19 surges over the winter months [14]. In previous work, we identified that 23% of surveyed UK HCWs were hesitant about receiving initial doses of the SARS-CoV-2 vaccine. We have shown that certain demographic factors including younger age, female sex and Black ethnicity are associated with SARS-CoV-2 vaccine hesitancy [15, 16] and that a lack of trust in official sources of vaccine information was associated with greater odds of persistent SARS-CoV-2 vaccine hesitancy in UK HCWs [17]. A small US survey study determined the proportion of HCWs that would be hesitant about receiving a hypothetical yearly SARS-CoV-2 booster to be 16.4% and that hesitancy about receiving the initial doses of SARS-CoV-2 vaccine was associated with hesitancy for receiving an annual vaccination [18]. However, there are no studies which have examined views towards receiving regular SARS-CoV-2 vaccination in UK HCWs.

With this in mind, we conducted a cross-sectional analysis to quantify hesitancy for receiving regular SARS-CoV-2 vaccination in a cohort of UK HCWs using data from the United Kingdom Research study into Ethnicity And COVID-19 outcomes in Healthcare workers (UK-REACH) longitudinal cohort study. We also sought to determine the associations between sociodemographic and occupational factors with hesitancy for receiving regular SARS-CoV-2 vaccination and to examine the relationship between trusted sources of vaccine information and exposure to information advocating against vaccination with hesitancy for receiving regular vaccination.

## Methods

### Overview

UK-REACH is a programme of work which aims to determine the impact of the COVID-19 pandemic on UK HCWs and establish whether, and to what degree, this differs according to ethnicity. This analysis uses data from the baseline questionnaire (administered between December 2020 and March 2021) and the first follow-up questionnaire (administered between April and June 2021) of the prospective nationwide cohort study. Details of the study design, sampling and measures included in the baseline questionnaire can be found in the study protocol [19] and the data dictionary (https://www.uk-reach.org/data-dictionary).

### Study population

We recruited individuals aged 16 years or over, living in the UK and employed as HCWs or ancillary workers in a healthcare setting and/or registered with one of seven major UK professional regulatory bodies (for a list of participating regulators, see Additional file 1: Supplementary text).

### Recruitment

Recruitment is described in detail in the study protocol [19] and in previous publications [20–22]. In brief, participating UK healthcare regulators (for a list of regulators, see Additional file 1: Supplementary text) sent emails to their registrants informing them of the study. The sample was supplemented by direct recruitment through participating healthcare trusts and advertising on social media and in newsletters. Those interested could access the study website, read the participant information sheet and provide online consent, after which they could access the baseline questionnaire. Invitations to complete the second questionnaire were emailed to all consented participants.

### Outcome measures

Our primary outcome was hesitancy for regular SARS-CoV-2 vaccination, as determined by the answer given to the following question on the second questionnaire ‘Would you be willing to have a COVID-19 vaccination regularly (for example, like the flu vaccination programme) if it was advised?’. Participants could answer on a 4-point scale from ‘definitely yes’ to ‘definitely no’; those selecting options other than ‘definitely yes’ were coded as hesitant (see Additional file 2: Table S1 for details).

### Covariates

We selected potential risk factors for regular SARS-CoV-2 vaccine hesitancy based on our previous work and with reference to the literature [17, 20, 21, 23]. These comprised the following:

- Demographic characteristics (age, sex, ethnicity). Ethnicity was categorised using the 5 broad Office for National Statistics (ONS) categories (White, Asian, Black, Mixed, Other) [24]
- Occupation: collapsed into five categories ‘medical’; ‘nurses, nursing associates, midwives’; ‘allied health professional (including pharmacists, healthcare scientists, ambulance workers and those in optical roles)’; ‘dental’; and ‘administrative/estates/others’
- Deprivation in residential areas: as determined by the index of multiple deprivation (IMD) [25]
- Previous history of COVID-19 as determined by self-reported polymerase chain reaction (PCR) or serology results (categorised as never tested, negative and positive)
- Influenza vaccination status in the two seasons preceding the second questionnaire
- Trust in one’s employing organisation to deal with a concern about unsafe clinical practice
- Total score on questionnaire items designed to assess belief in COVID-19 ‘conspiracy theories’
- Total score on questionnaire items designed to assess pro-vaccine attitudes
- Perceived risk of requiring hospital treatment if COVID-19 is contracted

Variables indicating trust in vaccine information sources and whether or not a participant had received information advocating against vaccination from a particular source were derived from the questionnaire items ‘Which sources of information do you trust to provide you with information about COVID-19 vaccination?’ and ‘Have any of the following groups of people suggested to you that you should not have the COVID-19 vaccine?’, respectively. Participants could select from a list of options and could select multiple options. Therefore, we generated binary (‘dummy’) variables for each information source to compare those who selected a particular option with those who did not.

The majority of these covariates are derived from responses to items on the second questionnaire or by combining information from both questionnaires. IMD and the COVID-19 conspiracies score are derived from information on the first questionnaire. A more detailed description of each variable, how it was derived from questionnaire responses and the questionnaire from which it was derived can be found in Additional file 3: Table S2.

### Statistical analysis

**Fig. 1:**
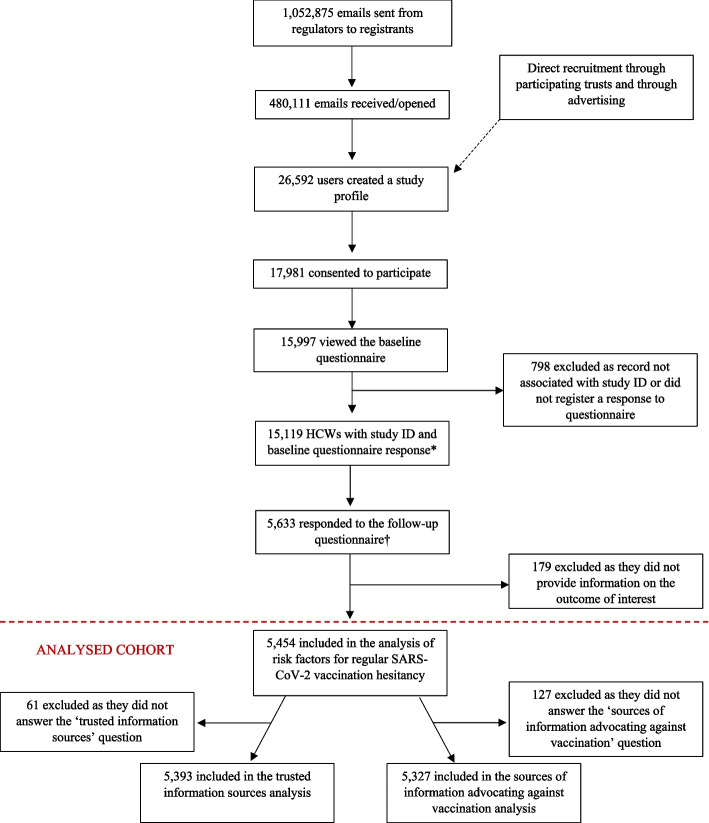
The association between trusted vaccine information sources and sources of information advocating against vaccination with hesitancy for receiving regular SARS-CoV-2 vaccination. *corresponds to a response rate of 57.1% of those who registered/created a profile on the study website (and 84.5% of those who consented, 1.4% of those who were sent an email and 3.2% of those who opened the email). †corresponds to a response rate of 37.1% of those who responded to the baseline questionnaire and 21.2% of those who registered/created a profile on the study website (and 31.3% of those who consented, 0.5% of those who were sent an email and 1.2% of those who opened the email)

We excluded those with missing data for the outcome of interest, including those who answered ‘prefer not to answer’ to the relevant question, from all analyses. We excluded those who did not answer the questions relating to trusted information sources or information sources advocating against vaccination from the relevant analyses (see Fig. 1 for further details).

We summarised categorical variables as frequency and percentage and non-normally distributed continuous variables as median (interquartile range [IQR]). We used univariable and multivariable logistic regression to determine unadjusted and adjusted associations of the variables described above with willingness to accept regular SARS-CoV-2 vaccination (the multivariable model was adjusted for all hypothesised risk factors/protective factors).

Multiple imputation by chained equations was used to impute missing data in all logistic regression models. The imputation model contained all variables bar those being imputed, including the outcome measure. Where a measure appeared in the first and second questionnaires, both measures were included in the imputation models, and the measure from the second questionnaire was used in the analysis. Rubin’s Rules were used to combine the parameter estimates and standard errors from 10 imputations into a single set of results [26]. Although indices of deprivation are available for UK countries outside England, it is recognised that these are not directly comparable with English IMD [27]. We therefore elected to code IMD as missing for those outside England and impute the missing information. To investigate the impact of imputation on our results, we also conducted a complete case analysis and compared the results to those derived from imputed data.

In the analyses relating to trusted information sources and sources of information advocating against vaccination, we examined the association of the binary variables with the outcome measure (hesitancy about receiving regular SARS-CoV-2 vaccine) adjusted for age, sex, ethnicity and occupation and presented the results as a figure showing adjusted odds ratios and 95% confidence intervals.

To examine the effects of participation bias, we compared the key demographic and occupational parameters in those that responded to the second questionnaire and those that did not using the Wilcoxon rank-sum test for continuous variables and the chi-squared test for categorical variables.

All analyses were conducted using Stata 17 (StataCorp. 2021; Stata Statistical Software: Release 17; College Station, TX; StataCorp LLC).

### Ethical approval

The study was approved by the Health Research Authority (Brighton and Sussex Research Ethics Committee; ethics reference: 20/HRA/4718). All participants gave written informed consent.

### Involvement and engagement

We worked closely with a professional expert panel comprising an ethnically and occupationally diverse group of HCWs as well as with national and local organisations (see study protocol).^1^

### Role of the funding source

The funders had no role in the study design, data collection, data analysis, interpretation or writing of the report.

## Results

### Cohort recruitment and formation of the analysed sample

Figure 1 shows the formation of the analysed sample. A total of 15,199 HCWs responded to the baseline questionnaire, of whom 5633 responded to the second questionnaire. A total of 5454 of these HCWs provided data on the outcome of interest and were included in the analysis of hesitancy risk factors. A total of 61 participants did not provide information on trusted vaccine information sources, and 127 did not provide information on sources of information advocating against vaccination and were excluded from the relevant analyses (see Fig. 1).

Second questionnaire responders were slightly older than non-responders (median [IQR] age 46 [35–55] vs 44 [34–53], respectively, *p* < 0.001). The proportion of participants from ethnic minority groups was similar amongst responders and non-responders. Although the proportion of White participants was higher amongst responders than non-responders (72.9% vs 55.9%, respectively), this may be due to White participants being less likely to provide information on their ethnicity in the baseline questionnaire. There were slight, but significant, differences in the proportion of occupational groups who responded to the second questionnaire compared to those that did not (Additional file 4: Table S3).

### Description of the analysed cohort

Table 1 contains a description of the cohort stratified by selected predictor variables. The Median (IQR) age was 46 (35–55) years. The majority of the cohort were female (74.9%), and 26.7% were from ethnic minority groups (17.3% Asian, 3.3% Black, 4.1% mixed and 1.8% others).

**Table 1:** Description of cohort

**Variable**	**Description,** **total (*N* = 5454)**
**Age**, med (IQR)	46 (35–55)
Missing	25 (0.5)
**Sex**	
Male	1360 (24.9)
Female	4087 (74.9)
Missing	7 (0.1)
**Ethnicity**	
White	1317 (73.3)
Asian	945 (17.3)
Black	182 (3.3)
Mixed	226 (4.1)
Others	99 (1.8)
Missing	2 (0.0)
**Occupation**	
Medical	1317 (24.2)
Nurses, NA, midwives	1122 (20.6)
AHPs^a^	2220 (40.7)
Dental	314 (5.8)
Admin/estates/others	325 (6.0)
Missing	156 (2.9)
**Index of multiple deprivation quintile**	
1 (most deprived)	429 (7.9)
2	824 (15.1)
3	995 (18.2)
4	1207 (22.1)
5 (least deprived)	1398 (25.6)
Missing	601 (11.0)
**Previous COVID-19 (by PCR or serology)**	
Never tested	459 (8.4)
Tested negative	3630 (66.6)
Tested positive	1332 (24.4)
Missing	33 (0.6)
**Trust in organisation (to address concern about unsafe clinical practice)**	
Does not trust organisation	1357 (24.9)
Trusts organisation	3593 (65.9)
Missing	504 (9.2)
**Pro-vaccine score (scale 4–20), med (IQR)**	16 (14–17)
Missing	28 (0.5)
**Number of influenza vaccinations in previous 2 seasons**	
0	740 (13.6)
1	862 (15.8)
2	3595 (65.9)
Missing	257 (4.7)
**COVID-19 conspiracies score (scale 6–24), med (IQR)**	8 (7–10)
Missing	247 (4.5)
**Personal risk of being hospitlalised with COVID-19 in the next 6 months (scale 0–100), med (IQR)**	10 (3–25)
Missing	57 (1.1)

All data are *n* ( %) unless otherwise stated. Percentages are computed column-wise.

*AHP* allied health professional, *COVID-19* coronavirus disease 2019, *IQR* interquartile range, *med* median, *NA* nursing associate, *PCR* polymerase chain reaction

^a^Also includes pharmacists, healthcare scientists, ambulance workers and those in optical roles

### Univariable analysis of factors associated with hesitancy for receiving regular SARS-CoV-2 vaccination

Table 2 contains a description of the cohort stratified by the outcome measure and unadjusted odds ratios for the association of covariates with the outcome measure (hesitancy to receive regular SARS-CoV-2 vaccination). Overall, 1282 (23.5%) of respondents indicated hesitancy for the regular SARS-CoV-2 vaccine. Odds of hesitancy decreased as age increased (0.76, 95%CI 0.72–0.80, *p* < 0.001 [per decade increase in age]). Females were more likely to be hesitant than males (OR 1.27, 95%CI 1.09–1.47, *p* = 0.002). Black and Asian HCWs are more likely to be hesitant than White HCWs (OR 3.42, 95%CI 2.53–4.62 and OR 1.45, 95%CI 1.24–1.71, respectively, *p* < 0.001 for both).

**Table 2:** Comparison of the hesitant and non-hesitant cohorts with unadjusted and adjusted odds ratios for an outcome of hesitancy for receiving regular SARS-CoV-2 vaccination

Variable	Not hesitant about receiving regular SARS-CoV-2 vaccine, 4172 (76.5)	Hesitant about receiving regular SARS-CoV-2 vaccine, 1282 (23.5)	Unadjusted OR (95%CI)	*P* value	Adjusted OR (95%CI)	*P* value
**Age**, med(IQR)^a^	47 (36–56)	41 (33–52)	0.76 (0.72–0.80)	< 0.001	0.78 (0.73–0.83)	< 0.001
Missing	18 (0.4)	7 (0.6)				
**Sex**						
Male	1083 (26.0)	277 (21.6)	Ref	–	Ref	–
Female	3086 (74.0)	1001 (78.1)	1.27 (1.09–1.47)	0.002	1.15 (0.95–1.37)	0.15
Missing	3 (0.1)	4 (0.3)				
**Ethnicity**						
White	3155 (75.6)	845 (65.9)	Ref	–	Ref	–
Asian	680 (16.3)	265 (20.7)	1.45 (1.24–1.71)	< 0.001	1.14 (0.93–1.40)	0.22
Black	95 (2.3)	87 (6.8)	3.42 (2.53–4.62)	< 0.001	2.60 (1.80–3.72)	< 0.001
Mixed	168 (4.0)	58 (4.5)	1.29 (0.95–1.75)	0.11	1.30 (0.91–1.85)	0.15
Others	74 (1.8)	25 (2.0)	1.26 (0.80–1.99)	0.33	1.10 (0.64–1.90)	0.72
Missing	0 (0.0)	2 (0.2)				
**Occupation**						
Medical	1081 (25.9)	236 (18.4)	Ref	–	Ref	–
Nurses, NA, midwives	869 (20.8)	253 (19.7)	1.32 (1.09–1.61)	0.005	1.07 (0.83–1.38)	0.62
AHPs^†^	1657 (39.7)	563 (43.9)	1.55 (1.31–1.84)	< 0.001	1.16 (0.94–1.44)	0.17
Dental	211 (5.1)	103 (8.0)	2.25 (1.71–2.95)	< 0.001	0.89 (0.63–1.24)	0.48
Admin/estates/others	242 (5.8)	83 (6.5)	1.58 (1.18–2.12)	0.002	1.17 (0.82–1.66)	0.38
Missing	112 (2.7)	44 (3.4)				
**Index of multiple deprivation quintile**						
1 (most deprived)	298 (7.1)	131 (10.2)	1.40 (1.10–1.80)	0.007	0.97 (0.73–1.30)	0.86
2	587 (14.1)	237 (18.5)	1.28 (1.04–1.58)	0.02	1.11 (0.85–1.45)	0.44
3	761 (18.2)	234 (18.3)	Ref	–	Ref	–
4	945 (22.7)	262 (20.4)	0.88 (0.72–1.08)	0.22	0.95 (0.75–1.20)	0.67
5 (least deprived)	1113 (26.7)	285 (22.2)	0.81 (0.67–0.98)	0.03	0.92 (0.73–1.15)	0.45
Missing	468 (11.2)	133 (10.4)				
**Previous COVID-19 (by PCR or serology)**						
Never tested	358 (8.6)	101 (7.9)	1.02 (0.81–1.29)	0.84	0.73 (0.56–0.96)	0.02
Tested negative	2836 (68.0)	794 (61.9)	Ref	–	Ref	–
Tested positive	958 (23.0)	374 (29.2)	1.38 (1.20–1.60)	< 0.001	1.33 (1.13–1.57)	0.001
Missing	20 (0.5)	13 (1.0)				
**Trust in employing organisation (to address concern about unsafe clinical practice)**						
Does not trust organisation	974 (23.4)	383 (29.9)	Ref	–	Ref	–
Trusts organisation	2827 (67.8)	766 (59.8)	0.70 (0.61–0.81)	< 0.001	0.80 (0.68–0.94)	0.005
Missing	371 (8.9)	133 (10.4)				
**Pro-vaccine score (scale 4–20)**^b^**, med (IQR)**	16 (15–18)	14 (12–16)	0.73 (0.71–0.75)	< 0.001	0.77 (0.75–0.79)	< 0.001
Missing	14 (0.3)	14 (1.1)				
**Number of influenza vaccinations in previous 2 seasons**						
0	323 (7.7)	417 (32.5)	Ref	–	Ref	–
1	610 (14.6)	252 (19.7)	0.32 (0.26–0.40)	< 0.001	0.36 (0.29–0.45)	< 0.001
2	3046 (73.0)	549 (42.8)	0.14 (0.12–0.17)	< 0.001	0.18 (0.14–0.21)	< 0.001
Missing	193 (4.6)	64 (5.0)				
**COVID-19 conspiracies score (scale 6–24)**^b^**, med (IQR)**	8 (7–10)	9 (8–11)	1.25 (1.22–1.29)	< 0.001	1.06 (1.02–1.10)	0.002
Missing	178 (4.3)	69 (5.4)				
**Personal risk of being hospitalised with COVID-19 in the next 6 months (scale 0–100)**^c^	10 (3–25)	10 (2–25)	0.99 (0.96–1.02)	0.53	0.97 (0.93–1.00)	0.06
Missing	36 (0.9)	21 (1.6)				

All data in the left two columns are *n* (%) unless otherwise stated. Percentages are computed column-wise other than the total proportion of the hesitant and non-hesitant cohorts which are computed row-wise

*AHP* allied health professional, *COVID-19* coronavirus disease 2019, *IQR* interquartile range, *med* median, *NA* nursing associate, *PCR* polymerase chain reaction, *Ref* reference category for regression analyses

^a^Odds ratios and adjusted odds ratios are per decade increase in age

^b^For details on the derivation of the score, please see Additional file 3: Table S2

^c^Odds ratios and adjusted odds ratios are per 10-point increase in score

^†^Includes scientists and those in optical, ambulance and pharmacy roles

### Multivariable analysis of factors associated with hesitancy for receiving regular SARS-CoV-2 vaccination

#### Sociodemographic and occupational descriptors

Table 2 shows the results of the multivariable analyses. We found that older HCWs had lower odds of hesitancy for regular SARS-CoV-2 vaccination (aOR 0.78, 0.73–0.83, *p* < 0.001 [per decade increase in age]). Black HCWs had over two and a half times the odds of hesitancy compared to White HCWs (aOR 2.60, 95%CI 1.80–3.72, *p* < 0.001). Asian HCWs were not significantly more likely to be hesitant than White HCWs after adjustment.

#### Attitudes and behaviours

Those who received two influenza vaccinations over the past two seasons were around five times less likely to be hesitant about receiving regular SARS-CoV-2 vaccination than those who had not been vaccinated against influenza over this time period (aOR 0.18, 95%CI 0.14–0.21, *p* < 0.001). As pro-vaccine scores increased, the odds of hesitancy decreased (aOR 0.77, 95%CI 0.75–0.79, *p* < 0.001 for each point increase) and those who trusted their employing organisation to deal with concerns about the unsafe clinical practice were less likely to be hesitant compared to those who did not (aOR 0.80, 95%CI 0.68–0.94, *p* = 0.005). The odds of hesitancy increased with an increasing score on a COVID-19 ‘conspiracies score’ (aOR 1.06, 95%CI 1.02–1.10, *p* = 0.002 for each point increase). HCWs who had previously tested positive for SARS-CoV-2 were more likely to be hesitant compared to those who had tested negative (aOR 1.33, 95%CI 1.13–1.57, *p* = 0.001).

### Association of vaccine information sources and hesitancy for receiving regular SARS-CoV-2 vaccination

**Fig. 2:**
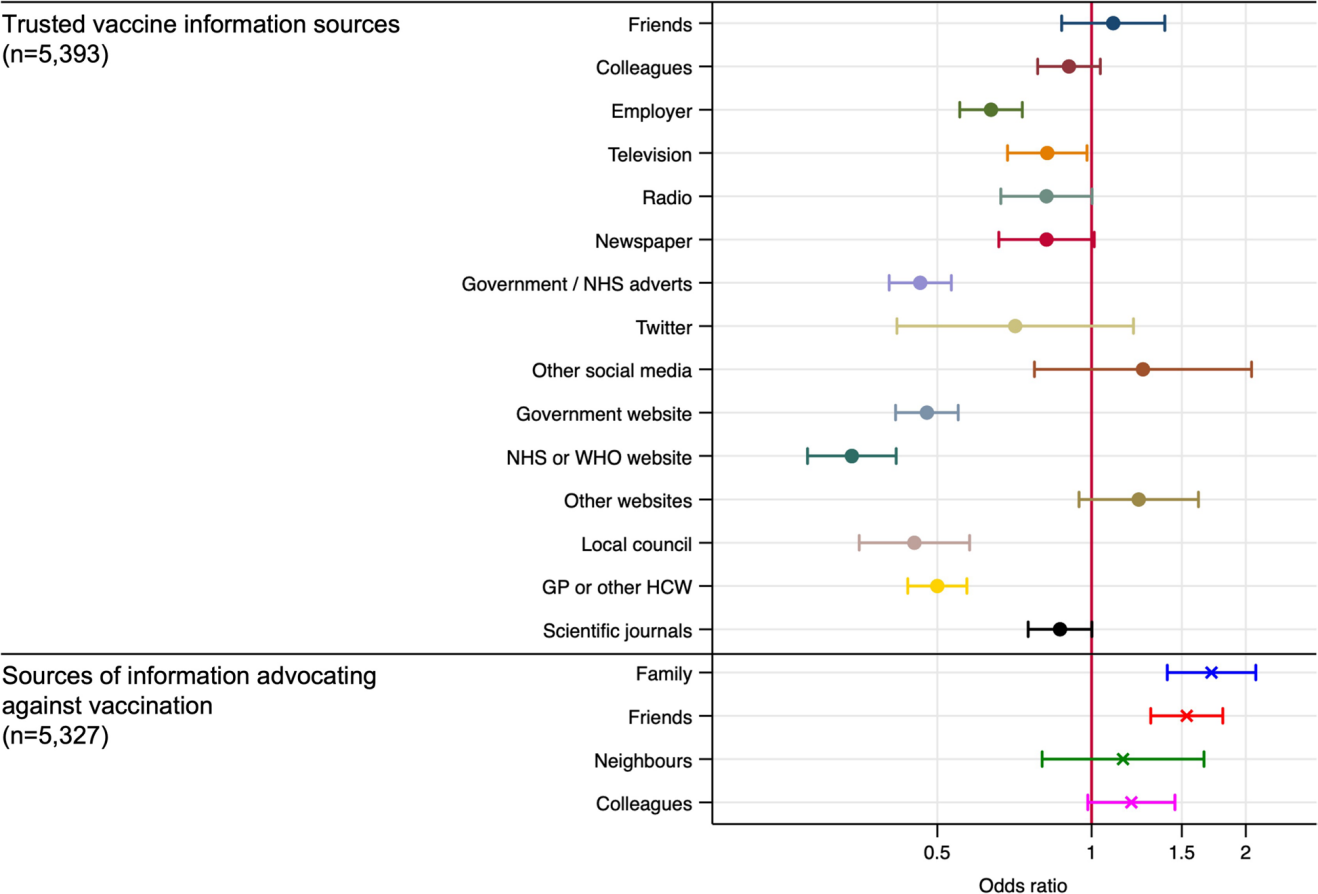
Shows the association between sources of vaccine related information that a participant indicated they trusted, and sources of information advocating against vaccination that a participant indicated they had been exposed to, with hesitancy for regular SARS-CoV-2 vaccination. All information sources variables are binary, those that indicated they trusted an information source are compared to those who indicated they did not. Those who had been in receipt of information advocating against source are compared to those who indicated they did not. Those who had been in receipt of information advocating against occupation. GP – General practitioner; HCW – Healthcare worker

Figure 2 shows the association between trusted sources of vaccine information and exposure to sources of information advocating against vaccination with hesitancy for regular SARS-CoV-2 vaccination. Those who indicated trust in government or NHS/WHO websites (government website: aOR 0.48, 95%CI 0.41–0.55, *p* < 0.001; NHS/WHO website: aOR 0.34, 95%CI 0.28–0.42, *p* < 0.001) or adverts (government/NHS adverts: aOR 0.46, 95%CI 0.40–0.53, *p* < 0.001) were less than half as likely to be hesitant than those who did not. Those who trusted other HCWs (aOR 0.50, 95%CI 0.44–0.57, *p* < 0.001) or their employers (aOR 0.64, 95%CI 0.55–0.73, *p* < 0.001) were also less likely to be hesitant about receiving regular SARS-CoV-2 vaccination. Receiving information advocating against vaccination from family members (aOR 1.71, 95%CI 1.41–2.09, *p* < 0.001) or friends (aOR 1.53, 95%CI 1.30–1.80, *p* < 0.001) was associated with increased odds of hesitancy for receiving regular SARS-CoV-2 vaccination.

### Sensitivity analysis

In an analysis using complete cases only, significant findings remained unchanged (see Additional file 5: Table S4).

## Discussion

This is the first study to evaluate hesitancy to receive regular SARS-CoV-2 vaccination in the UK. In our study of HCWs, around a quarter of respondents were hesitant about having regular vaccinations against SARS-CoV-2. This is similar to the results from a smaller US study which demonstrated that 83.6% of HCWs would accept a hypothetical yearly SARS-CoV-2 vaccination [18]. In our cohort, individuals who were younger, from Black ethnic groups, who had a previous episode of COVID-19, who had fewer influenza vaccinations in the last two seasons, and who scored higher on a COVID-19 conspiracy beliefs scale, were more likely to be hesitant about receiving regular vaccination. HCWs who reported trusting official information sources (e.g. government or NHS sources) for vaccine-related information were less likely to be hesitant and those who had received information advocating against vaccination from family and friends were more likely to be hesitant to receive regular SARS-CoV-2 vaccination.

Our findings relating to ethnic differences in hesitancy are particularly concerning given the higher likelihood of infection and severe COVID-19 in ethnic minority groups [15]. The reasons underlying SARS-CoV-2 vaccine hesitancy in ethnic minority groups are complex and multi-faceted. Previous work has suggested that a lack of trust in the government and pharmaceutical companies due to historic unethical research practices in vaccine studies, institutional racism, lack of confidence in the vaccine and/or increased concern of side effects following vaccination may contribute [15, 28–30]. Indeed, previous work in the UK-REACH cohort has identified ethnic differences in trust in employing organisations, pro-vaccine scores and COVID-19 conspiracy scores [20]. Our work highlights the importance of trust (in employer, in the NHS and in the government) by demonstrating that those who trust their employing organisation (to address a concern about unsafe clinical practice) and that those who trust in government and NHS sources of vaccine information are less likely to be hesitant about receiving a regular SARS-CoV-2 vaccination. Although the UK has abandoned mandatory SARS-CoV-2 vaccines for HCWs, some European countries are still implementing mandatory vaccination for certain, higher-risk groups [31, 32]. In the context of our findings, mandatory vaccinations would be expected to disproportionately impact younger HCWs and those from Black ethnic groups. This could lead to negative sequelae in terms of perceived workplace discrimination, morale and workforce attrition [21, 33]. In the community, mandatory vaccination could heighten mistrust of the government and healthcare providers and act as a barrier to accessing healthcare, exacerbating health inequalities.

HCWs who have previously tested positive for COVID-19 are more likely to be hesitant about receiving regular SARS-CoV-2 vaccination than those who tested negative. Younger HCWs are also more likely to be hesitant. These findings may be due to a lower perceived risk of infection or severe illness due to younger age or having previously suffered minor symptoms from COVID-19 (although, in our study, we did not find an association between perceived risk of hospitalisation with COVID-19 and hesitancy). Emerging evidence suggests that re-infection risk increases with time, especially with omicron and its subvariants [22, 23]. Whilst reinfection does not necessarily result in severe disease, reinfected HCWs may emit large quantities of virus for limited periods of time [34]. In a healthcare setting, this could result in transmission to vulnerable patients who are at risk of COVID-19 mortality, such as the elderly, frail and those with multiple comorbidities. Education programmes focused on the relation between receiving vaccines and preventing transmission to others may be valuable within these HCW subcohorts to improve uptake moving forwards.

We found that those who received influenza vaccination in the preceding two seasons were over five times less likely to be hesitant about receiving regular SARS-CoV-2 vaccination. This important finding may represent acceptance amongst people who already engage in regular vaccination programmes and therefore are likely to trust vaccines to protect themselves and their patients against disease. Our findings suggest that healthcare employers could use previously acquired data on influenza vaccine uptake to proactively identify staff groups at risk of SARS-CoV-2 vaccine hesitancy, provided such analysis was carried out on pseudonymised or aggregate level data and used to identify groups at risk rather than target individuals. Specifically, it might be considered that if particular clinical or non-clinical areas with low influenza vaccine uptake are identified then SARS-CoV-2 vaccination could be delivered in these areas (rather than via appointment at a vaccine hub or with the occupational health team) to improve access to vaccination. Awareness campaigns on the dangers of COVID-19 and the safety of vaccination could also be targeted towards such areas. Both of these interventions have previously been shown to be effective in improving influenza vaccine uptake in HCW [35].

We have identified an association between the sources of vaccine information that HCWs trust and hesitancy about receiving regular SARS-CoV-2 vaccination. These findings, together with the correlation between COVID-19 conspiracy belief score and odds of regular vaccine hesitancy, underscore the importance of trust in the government and healthcare organisations for influencing decisions relating to vaccine uptake. This has been demonstrated previously both for vaccine uptake in general [36] and specifically for uptake of the initial doses of the SARS-CoV-2 vaccine [20]. Our results indicate that, in the longer term, building trust in these organisations, particularly amongst groups likely to exhibit vaccine hesitancy (such as those from ethnic minority groups) is of paramount importance. The specific changes required to build trust in such organisations will vary depending on the group in question. For example, in HCW from ethnic minority groups whose trust in their employing organisation may be undermined by an increased risk of discrimination and harassment at work [37], structural changes are required to support staff in speaking up about discrimination, enabling career progression of ethnic minority staff and promoting diversity in senior leadership roles [38]. Instituting such changes is complex and thus may take considerable time and commitment. In the shorter term, trust in vaccination both in HCW and in the general population may be improved by active engagement with communities and mobilisation of trusted networks (faith leaders, community champions) to open dialogues around vaccine uptake in an effort to emphasise the positive effects of vaccination and allay any concerns [39, 40]. It is of note that the impact of a HCW’s social network on decisions regarding regular SARS-CoV-2 vaccination is highlighted in our current work, where we demonstrate that those who had received information advocating against vaccination from family and friends were more likely to be hesitant about receiving regular vaccination. This would suggest that community-based interventions might be effective at improving vaccine uptake in HCWs.

Our work has some limitations. As with any consented cohort study, our results may be affected by selection and participation biases. This could lead to the over-representation of people who are more likely to hold pro-vaccination views. Selection bias in the baseline UK-REACH cohort has been explored elsewhere [20], and in the current work, we provide a comparison of the occupational and demographic features of those who responded to the second questionnaire and those who did not to examine participation bias. Although there are differences between those that completed the second questionnaire and those that did not, these differences are small, and we do not anticipate this to have had a significant impact on our results. The majority (74.9%) of our cohort is female. Previous work has shown that female HCWs are more likely to be vaccine hesitant than male HCWs [20, 23, 41, 42]. However, the proportion of females in our sample is similar to the proportion of females in the NHS workforce (76.7% in 2021 [43]), and thus, we believe our sample is representative in terms of sex. We cannot predict how the uptake of vaccinations will be affected by policies implemented after the period of data collection, for example, removing the legal requirement to self-isolate when diagnosed with COVID-19 in the UK. Finally, the cross-sectional nature of the study means we cannot infer the direction of all the reported associations.

## Conclusions

We have identified factors associated with hesitancy to receive regular SARS-CoV-2 vaccinations and demonstrated an association between trusted vaccine information sources and sources of information advocating against vaccination with reported hesitancy for regular vaccination. Our findings should be used to improve (1) future interventional research studies aiming to develop and evaluate tools for reducing vaccine hesitancy in HCW and (2) resource planning and policies aimed at improving and maintaining SARS-CoV-2 vaccine uptake within healthcare organisations.

## Supplementary Information

**Additional file 1: Supplementary text.** List of participating healthcare regulators.

**Additional file 2: Table S1.** Derivation of binary outcome variable.

**Additional file 3: Table S2.** Derivation of variables used in the analysis from questionnaire data.

**Additional file 4: Table S3.** Comparison of key demographic and occupational parameters of second questionnaire responders and non-responders.

**Additional file 5: Table S4.** Unadjusted and adjusted odds ratios for an outcome of hesitancy for regular SARS-CoV-2 vaccination using complete cases (*n*=4030).

## Data Availability

To access data or samples produced by the UK-REACH study, the working group representative must first submit a request to the Core Management Group by contacting the UK-REACH Project Manager in the first instance. For ancillary studies outside of the core deliverables, the Steering Committee will make the final decisions once they have been approved by the Core Management Group. Decisions on granting access to data/materials will be made within 8 weeks. Third-party requests from outside the project will require the explicit approval of the Steering Committee once approved by the Core Management Group. Note that should there be significant numbers of requests to access data and/or samples, then a separate Data Access Committee will be convened to appraise requests in the first instance.

## Abbreviations

95%CI: 95% Confidence interval
aOR: Adjusted odds ratio
COVID-19: Coronavirus disease 2019
HCW: Healthcare worker
IMD: Index of multiple deprivation
IQR: Interquartile range
NHS: National Health Service
ONS: Office for National Statistics
OR: Odds ratio
PCR: Polymerase chain reaction
SARS-CoV-2: Severe acute respiratory syndrome coronavirus-2
UK: United Kingdom
UK-REACH: United Kingdom Research study into Ethnicity And COVID-19 outcomes in Healthcare workers
WHO: World Health Organisation
