# Supplementary material for: Hesitancy for receiving regular SARS-CoV-2 vaccination in UK healthcare workers: a cross-sectional analysis from the UK-REACH study
Source: BMC Med. 2022 Oct 10;20:386. doi: 10.1186/s12916-022-02588-7 (PMC9548389; doi:10.1186/s12916-022-02588-7)
Supplement: Supplementary file 1 — Additional file 1: Supplementary text. List of participating healthcare regulators. [file 12916_2022_2588_MOESM1_ESM.docx]

**Additional file 1: Supplementary text.** List of participating healthcare regulators.

- The General Medical Council (GMC)
- The Nursing and Midwifery Council (NMC)
- The General Dental Council (GDC)
- The Health and Care Professions Council (HCPC)
- The General Optical Council (GOC)
- The General Pharmaceutical Council (GPC)
- The Pharmaceutical Society of Northern Ireland (PSNI)
