# Supplementary material for: Hesitancy for receiving regular SARS-CoV-2 vaccination in UK healthcare workers: a cross-sectional analysis from the UK-REACH study
Source: BMC Med. 2022 Oct 10;20:386. doi: 10.1186/s12916-022-02588-7 (PMC9548389; doi:10.1186/s12916-022-02588-7)
Supplement: Supplementary file 2 — Additional file 2: Table S1. Derivation of binary outcome variable. [file 12916_2022_2588_MOESM2_ESM.docx]

**Additional file 2: Table S1.** Derivation of binary outcome variable

| Would you be willing to have a COVID-19 vaccination regularly (for example, like the flu vaccination programme) if it was advised? | OUTCOME | N(%)  Total = 5,454 |
| --- | --- | --- |
| Definitely yes | Not hesitant | 4172 (76.5) |
| Probably yes | Hesitant | 1022 (18.7) |
| Probably no | Hesitant | 177 (3.3) |
| Definitely no | Hesitant | 83 (1.5) |

Supplementary Table 1 shows how the binary outcome variable was derived from the raw data and gives the frequency and percentage of the participants in the analysed cohort who selected each option. Participants could also select ‘Prefer not to answer’. If this option was selected then participants were excluded from the analysis. Prefer not to answer was selected by 13 participants.
