# Supplementary material for: Hesitancy for receiving regular SARS-CoV-2 vaccination in UK healthcare workers: a cross-sectional analysis from the UK-REACH study
Source: BMC Med. 2022 Oct 10;20:386. doi: 10.1186/s12916-022-02588-7 (PMC9548389; doi:10.1186/s12916-022-02588-7)
Supplement: Supplementary file 3 — Additional file 3: Table S2. Derivation of variables used in the analysis from questionnaire data. [file 12916_2022_2588_MOESM3_ESM.docx]

**Additional file 3: Table S2.** Derivation of variables used in the analysis from questionnaire data

| **Variable** | **Description** |
| --- | --- |
| **Age** | Continuous variable. Age in years. Derived from date of birth entered by participants at registration. |
| **Sex** | Binary variable. Participants were asked their sex assigned at birth. |
| **Ethnicity** | Categorical variable. Participants were asked to select their ethnicity from a list of the 18 Office for National Statistics categories:  Asian/Asian British – Indian  Asian/Asian British – Pakistani  Asian/Asian British – Bangladeshi  Asian/Asian British – Chinese  Asian/Asian British - Any other Asian background  Black/African/Caribbean/Black British - African  Black/African/Caribbean/Black British – Caribbean  Black/African/Caribbean/Black British - Any other Black/African/Caribbean background Mixed/Multiple ethnic groups - White and Black Caribbean  Mixed/Multiple ethnic groups - White and Black African  Mixed/Multiple ethnic groups - White and Asian  Mixed/Multiple ethnic groups - Any other Mixed/multiple ethnic background  White - English/Welsh/Scottish/Northern Irish/British  White – Irish  White - Gypsy or Irish Traveller  White - Any other white background  Other ethnic group – Arab  Other ethnic group - Any other ethnic background  These were categorised into the 5 broader Office for National Statistics ethnicity categories (Asian, Black, Mixed, White, Other). |
| **Occupation** | Categorical variable. Participants were asked to select their main job/role. Categorised as below:  **Doctor or medical support** - Doctor, Advanced Critical Care Practitioner, Anaesthesia associate, Surgical Care Practitioner, Other medical associate  **Nurse, NA or Midwife -**  Advanced Nurse Practitioner, Healthcare assistant, Maternity support worker, Midwife, Nurse, Nursing Associate, Other nursing and midwifery role,  **Allied Health Professional (including pharmacists, ambulance workers and those in optical roles)** - Arts therapist, Biomedical scientist, Chiropodist/Podiatrist, Clinical scientist, Dietician, Hearing aid dispenser, Occupational therapist, Operating department practitioner, Orthoptist, Physiotherapist, Practitioner psychologist, Prosthetist / Orthotist, Radiographer, Speech and language therapist, Other Allied Health Professional role, Emergency medical , Paramedic , Other ambulance role, OT Support , Phlebotomist, Physiotherapy Assistant, Radiography Other clinical support role , Pharmacist , Pharmacy technician, Other pharmacy role, Optical - Dispensing optician, Optometrist, Other Optical role  **Dental -**  Clinical dental technician, Dental Hygienist, Dental nurse, Dental technician, Dentist, Other dental role  **Admin, estates or other –** Administration, Catering services, Domestic services, Estates services, Porter, Other |
| **Index of Multiple Deprivation (IMD) quintile** | Ordinal variable. Participants provided their residential postcode on registration for the study. This was used to determine the Index of Multiple Deprivation (the official measure of deprivation for small areas of England) in the area in which they live. The IMD ranks all areas in England based on 7 measures of deprivation and the ranks can be expressed as quintiles. Lower quintiles indicate more deprivation. Although Wales, Scotland and Northern Ireland have their own measures of deprivation, these are said not to be directly comparable to English IMD and therefore we elected to impute an ‘English IMD’ for residents of the these nations. |
| **History of COVID-19** | Categorical variable. Three levels: never tested, tested negative and tested positive. Participants were asked if they had ever had a positive polymerase chain reaction (PCR) or serology test for COVID-19. The value from the second questionnaire was taken unless this information was missing and the value from the first questionnaire was ‘tested positive’, in which case the participant was coded as ‘tested positive’. |
| **Number of influenza vaccinations in previous 2 seasons** | Ordinal variable. Participants were asked in the first questionnaire if they had received an influenza vaccine in the 2019 – 2020 season and in the 2020 – 2021 season. In the second questionnaire participants were asked if they had a received an influenza vaccine in the last four months. Those answering ‘yes’ to this question were coded as having received vaccine in the 2020 – 2021 season. |
| **Trust in employer (to address a concern about unsafe clinical practice)** | Binary variable (trusts employer vs does not trust employer). Participants were asked to indicate how much they agree with the following statement “I am confident that my organisation would address my concern [about unsafe clinical practice]”. Participants who agreed or strongly agreed with this statement were coded as trusting their employer. |
| **COVID-19 conspiracies score** | Continuous variable. Range 6 – 24. Participants were asked to assess the veracity of 6 statements relating to COVID-19: “Coronavirus was created in a laboratory”, “Most people in the UK have already had coronavirus without realising it”, “The current pandemic is part of a global effort to force everyone to be vaccinated whether they want to or not”, “The number of people reported as dying from coronavirus is being deliberately reduced or hidden by the authorities”, “The symptoms that most people blame on coronavirus appear to be linked to 5G network radiation”, “There is no hard evidence that coronavirus really exists”. Participants could answer on the following scale; “Definitely true” (4 points), “Probably true” (3 points), “Probably false” (2 points), “Definitely false” (1 point). The scores from each questionnaire item were summed. Participants who partially completed the ‘conspiracies’ items were coded based on the mean score from the number of items they had answered multiplied by 6. |
| **Pro-vaccine score** | Continuous variable. Range 4 – 20. Participants were asked to indicate how much they agreed with the following statements: “I can rely on vaccines to stop serious infectious diseases”, “Although most vaccines appear to be safe, there may be problems that we have not yet discovered”, “Authorities promote vaccination for financial gain, not for people's health”, “Being exposed to diseases naturally is safer for the immune system than being exposed through vaccination. Answers were on a five point ‘Likert’ scale (strongly disagree to strongly agree). Scores between 1 and 5 for each item were given based on whether the statement was supportive of vaccination or not. The scores from each questionnaire item were summed. Participants who partially completed the items were coded based on the mean score from the number of items they had answered multiplied by 4. |
| **Personal risk of being hospitalised with COVID-19 in the next 6 months** | Continuous variable. Range 1 – 100. Participants were asked “ If you do catch coronavirus, what do you think are your chances of needing hospital treatment? Please enter a value on a scale from 0 to 100, where 0 means there is no possibility that you will and 100 means that you definitely will.” |
| **Trusted vaccine information sources*** | Binary / Dummy variables. Participants were asked “Which sources of information do you trust to provide you with information about COVID-19 vaccination? (Please select all that apply).” Answers were selected from a list of 17 sources (as well as ‘not applicable’ and ‘prefer not to answer’). In analysis we combined those selecting “UK Government website” with “Welsh, Scottish or NI government website” and those selecting “NHS website” with “WHO website”. |
| **Sources of information advocating against vaccination*** | Binary / Dummy variables. Participants were asked “Have any of the following groups of people suggested to you that you should not have the COVID-19 vaccine? Select all that apply.” |

*For a full list of information sources that participants could select from, please see the UK-REACH data dictionary (<https://www.uk-reach.org/main/data-dictionary/>)
