# Supplementary material for: Hesitancy for receiving regular SARS-CoV-2 vaccination in UK healthcare workers: a cross-sectional analysis from the UK-REACH study
Source: BMC Med. 2022 Oct 10;20:386. doi: 10.1186/s12916-022-02588-7 (PMC9548389; doi:10.1186/s12916-022-02588-7)
Supplement: Supplementary file 4 — Additional file 4: Table S3. Comparison of key demographic and occupational parameters of second questionnaire responders and non-responders. [file 12916_2022_2588_MOESM4_ESM.docx]

**Table S3. Comparison of key demographic and occupational parameters of second questionnaire responders and non-responders**

|  | **Did not respond to second questionnaire**  **(n=9,486)** | **Responded to second questionnaire**  **(n=5,633)** | **P value*** |
| --- | --- | --- | --- |
| **Age** in years, med (IQR)  Missing, n(%) | 44 (34 – 53)  60 (0.6) | 46 (35 – 55)  26 (0.5) | <0.001 |
| **Sex,** n(%)  Male  Female  Missing | 2,299 (24.2)  7,149 (75.4)  38 (0.4) | 1,411 (25.1)  4,215 (74.9)  7 (0.1) | 0.3 |
| **Ethnicity**, n(%)  White  Asian  Black  Mixed  Other  Missing | 5,305 (55.9)  1,660 (17.5)  396 (4.2)  328 (3.5)  195 (2.1)  1,602 (16.9) | 4,106 (72.9)  984 (17.5)  197 (3.5)  236 (4.2)  103 (1.8)  7 (0.1) | <0.001 |
| **Occupation**, n(%)  Medical  Nurses, NA, Midwives  AHPs  Dental  Admin/estates/other  Missing | 2,139 (23.9)  1,883 (21.1)  3,894 (43.6)  601 (6.7)  419 (4.7)  550 (5.8) | 1,360 (24.9)  1,159 (21.2)  2,285 (41.8)  329 (6.0)  335 (6.1)  165 (2.9) | 0.001 |

*P values are derived from Wilcoxon rank-sum test for continuous variables and from chi-squared tests for categorical variables – tests were performed on complete cases.

Occupational differences between the two groups may be due to participants changing jobs between questionnaires.

All data are n(%) unless otherwise stated. AHPs – Allied Health Professionals (also includes those in optical, ambulance and pharmacy roles)
