# Supplementary material for: Hesitancy for receiving regular SARS-CoV-2 vaccination in UK healthcare workers: a cross-sectional analysis from the UK-REACH study
Source: BMC Med. 2022 Oct 10;20:386. doi: 10.1186/s12916-022-02588-7 (PMC9548389; doi:10.1186/s12916-022-02588-7)
Supplement: Supplementary file 5 — Additional file 5: Table S4. Unadjusted and adjusted odds ratios for an outcome of hesitancy for regular SARS-CoV-2 vaccination using complete cases (n=4030). [file 12916_2022_2588_MOESM5_ESM.docx]

**Table S4. Unadjusted and adjusted odds ratios for an outcome of hesitancy for regular SARS-CoV-2 vaccination using complete cases (n=4,030)**

| **Variable** | **Unadjusted OR**  **(95% CI)** | **P value** | **Adjusted OR**  **(95% CI)** | **P value** |
| --- | --- | --- | --- | --- |
| **Age**, med(IQR)* | 0.77 (0.73 – 0.83) | <0.001 | 0.80 (0.74 – 0.86) | <0.001 |
| **Sex**  Male  Female | Ref  1.24 (1.04 – 1.48) | -  0.02 | Ref  1.11 (0.90 – 1.37) | -  0.32 |
| **Ethnicity**  White  Asian  Black  Mixed  Other | Ref  1.43 (1.19 – 1.73)  3.60 (2.51 – 5.16)  1.20 (0.83 – 1.73)  1.30 (0.73 – 2.32) | -  <0.001  <0.001  0.34  0.36 | Ref  1.15 (0.91 – 1.47)  3.09 (2.01 – 4.74)  1.15 (0.75 – 1.77)  1.17 (0.60 – 2.29) | -  0.25  <0.001  0.51  0.65 |
| **Occupation**  Medical  Nurses, NA, Midwives  AHPs†  Dental  Admin/estates/other | Ref  1.35 (1.07 – 1.69)  1.52 (1.25 – 1.85)  2.18 (1.58 – 3.01)  1.61 (1.17 – 2.21) | -  0.01  <0.001  <0.001  0.004 | Ref  1.15 (0.86 – 1.54)  1.14 (0.89 – 1.46)  0.79 (0.53 – 1.18)  1.21 (0.82 – 1.78) | -  0.35  0.30  0.25  0.34 |
| **Index of Multiple Deprivation quintile**  1 (most deprived)  2  3  4  5 (least deprived) | 1.44 (1.09 – 1.91)  1.19 (0.94 – 1.51)  Ref  0.86 (0.69 – 1.08)  0.84 (0.68 – 1.04) | 0.01  0.15  -  0.20  0.12 | 1.01 (0.73 – 1.40)  1.07 (0.81 – 1.41)  Ref  0.93 (0.73 – 1.21)  1.00 (0.78 – 1.28) | 0.94  0.63  -  0.60  0.99 |
| **Previous COVID-19 (by PCR or serology)**  Never tested  Tested negative  Tested positive | 1.08 (0.80 – 1.45)  Ref  1.41 (1.20 – 1.67) | 0.62  -  <0.001 | 0.82 (0.58 – 1.16)  -  1.36 (1.13 – 1.65) | 0.27  -  0.002 |
| **Trust in organisation (to address concern about unsafe clinical practice)**  Does not trust organisation  Trusts organisation | Ref  0.65 (0.56 – 0.77) | -  <0.001 | Ref  0.75 (0.63 – 0.91) | -  0.003 |
| **Pro-vaccine score (scale 4 – 20),** med (IQR) | 0.73 (0.70 – 0.75) | <0.001 | 0.77 (0.74 – 0.80) | <0.001 |
| **Number of influenza vaccinations in previous 2 seasons**  0  1  2 | Ref  0.27 (0.21 – 0.35)  0.13 (0.10 – 0.15) | -  <0.001  <0.001 | Ref  0.30 (0.23 – 0.39)  0.15 (0.12 – 0.19) | -  <0.001  <0.001 |
| **COVID-19 conspiracies score (scale 6 – 24),** med (IQR) | 1.27 (1.22 – 1.31) | <0.001 | 1.07 (1.02 – 1.11) | 0.003 |
| **Personal risk of being hospitalised with COVID-19 in the next 6 months (scale 0-100)** ‡ | 0.99 (0.95 – 1.02) | 0.43 | 0.95 (0.91 – 0.99) | 0.02 |

*odds ratios and adjusted odds ratios are per decade increase in age, † includes scientists and those in optical, ambulance and pharmacy roles, ‡ odds ratios and adjusted odds ratios are per 10 point increase in score.

Adjusted odds ratios are adjusted for all variables in the table.

AHP – Allied Health Professional; COVID-19 – coronavirus disease 2019; IQR – interquartile range; NA – nursing associate
